# LncRNA gadd7, increased in varicocele patients, suppresses cell proliferation and promotes cell apoptosis

**DOI:** 10.18632/oncotarget.23696

**Published:** 2017-11-24

**Authors:** Jun Zhao, Huan Li, Hao Deng, Li Zhu, Bingyu Zhou, MeiQiong Yang, QianRu Liu, GuoQun Luo, Yunxia Yang, WenMin Ma

**Affiliations:** ^1^ Assisted Reproductive Technology Center, Foshan Maternal and Child Health Care Hospital, Foshan, China

**Keywords:** gadd7, varicocele-related sperm impairment, male infertility

## Abstract

Varicocele-related sperm damages are usually caused by oxidative stresses. Growing evidence indicates that lncRNA growth arrested DNA-damage inducible gene 7 (gadd7) is involved in the regulation of the oxidative stress responses. In this study, we measured the expression level of gadd7 in the sperm and found that the expression of gadd7 was significantly up-regulated in patients with varicocele compared with the healthy control. The relative expression level of gadd7 was negatively correlated with the sperm count. Overexpression of gadd7 suppressed cell proliferation and promoted cell apoptosis in mouse spermatocyte-derived cell lines GC-1 and GC-2. Furthermore, the protein level of Bax was raised while Bcl2 expression was reduced after overexpression of gadd7. This work provides a potential novel insight for the varicocele-related sperm impairment and male infertility.

## INTRODUCTION

Infertility is considered a widespread public health problem and nearly half of infertility is caused by a male factor [1]. A varicocele might be noticed as a soft lump, which is an abnormal enlargement of the pampiniform venous plexus in the scrotum [2]. It is thought to result from abnormalities of the veins inside the testicles, leading to a backup of blood that cannot flow, which in turn causes swelling. About 15% of men in the general population have varicocele, while more than 40% of male infertility cases showed varicocele [3]. Varicocele may be one of the main contributors in male infertility due to the increased temperature and oxidative stress in scrotum [4]. Although the concept that a varicocele causes male subfertility has been around for more than 50 years now, the mechanisms by which a varicocele would destroy fertility have not yet been satisfactorily explained. Many genetic and epigenetic changes are found to be associated with varicocele-mediated male infertility [5], but the molecular pathological process is still far from understood. Therefore, a better identification of the biological markers of the male infertility is crucial, especially non-coding RNAs, as their gene expression pattern and function is not very clear.

Long non-coding RNAs (lncRNAs) are a class of non-coding RNAs that play critical functions in cellular response to stressful conditions [6]. Some lncRNAs were strongly up-regulated in the induction of oxidative stress by hydrogen peroxide treatment [7]. Deregulated lncRNAs have generated great interest in diagnosis and therapy for male infertility. Numerous studies focus on the complex network of lncRNAs and proteins in regulation of oxidative stress [8]. For example, lncRNA growth arrested DNA-damage inducible gene 7 (gadd7) binds to TAR DNA-binding protein (TDP-43) and promotes its dissociation from cyclin-dependent kinase 6 (Cdk6) mRNA, thus leading to degradation of Cdk6 mRNA [9]. Since Cdk6 is a key factor in the regulation of G1/S transition of the cell cycle, gadd7-mediated regulation contributes to the oxidative stress-mediated cell death.

Based on these previous results, we selected gadd7 as the possible marker for varicocele-mediated male infertility. We hypothesized that gadd7 may reduce cell proliferation and promote apoptosis.

In the present study, the expression patterns of gadd7 in the ejaculated spermatozoa of patients with varicocele were investigated by using Real-Time qPCR. Cell proliferation and apoptosis were measured after overexpression of gadd7 in the mouse germ cell lines GC-1 and GC-2. To the best of our knowledge, this is the first report showing the expression of gadd7 in varicocele-mediated male infertility and its potential functional roles.

## RESULTS

### Semen analysis

As shown in Table 1, the basic characteristics among varicocele grade II, varicocele grade III and healthy control group are comparable. No differences were noted in the mean age, volume, and pH among the three groups. However, the sperm count, motility, and vitality were significantly decreased in the varicocele groups. These sperm parameters were further decreased in the varicocele grade III group, suggesting that patients with varicocele have abnormal spermatogenesis and poor semen quality. The spermatozoa were then purified for RNA extraction and Real-Time qPCR.

**Table 1:** Patients and semen characteristics

Characteristics	Healthy control (n=28)	Varicocele		P value^*^
		Grade II (n=35)	Grade III (n=21)	
Age (years)	32.32±3.12	32.35±3.56	34.46±3.04	0.356
Volume (ml)	3.15±0.52	3.28±0.85	3.12±0.47	0.673
pH	7.38±0.12	7.41±0.24	7.38±0.32	0.731
Count (10 ^6^ /ml)	134.21±65.32	112.42±52.15	11.08±4.65	0.009
Percentage of motility	64.32±8.41	41.98±9.43	31.05±6.98	0.011
Percentage of vitality	87.45±9.33	50.14±11.32	28.80±7.32	0.008

^*^P value was calculated using one-way ANOVA. Data are presented as the mean ± standard deviation.

### gadd7 was up-regulated in the spermatozoa of patients with varicocele

**Figure 1:**
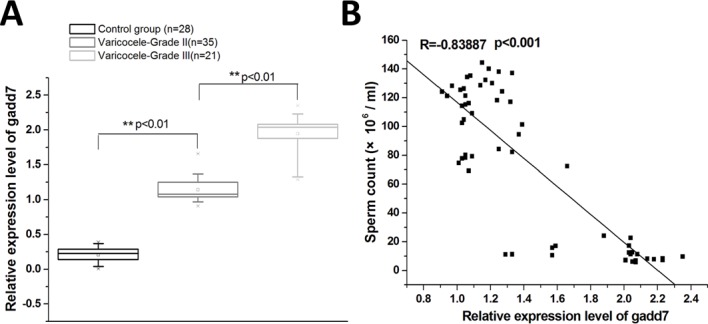
The expression of gadd7 in varicocele and healthy control groups The relative gadd7 expression levels were determined using Real-Time qPCR. GAPDH was used as an internal control. **(A)** qRT-PCR analysis of the expression of gadd7 in the ejaculated spermatozoa of varicocele groups or the healthy control. The expression differences were analyzed using independent samples *t*-test. **(B)** The relative expression level of gadd7 was negatively correlated with the sperm count. Pearson's coefficient correlation was used for expression correlation assay.

Since oxidative stress is one of the two major reasons for the varicocele-related spermatogenesis damage, we measured the expression of gadd7 which was reported to be involved in oxidative cellular stress. The relative expression levels of gadd7 were determined using Real-Time qPCR in a total of 56 patients with varicocele and 28 healthy controls. As shown in Figure 1A, gadd7 was up-regulated in varicocele groups compared to normal control group. The expression of gadd7 in the ejaculated sperm revealed significant increase in patients with varicocele grade III compared with varicocele grade II group. The relative expression level of gadd7 was negatively correlated with the sperm count (Figure 1B). Therefore, we investigated the functional roles of gadd7 in the following study.

### Overexpression of gadd7 inhibited cell proliferation

Mouse germ cell lines GC-1 and GC-2 were transfected with either pcDNA3.1-gadd7 or negative control plasmid and cultured in normal medium. Forty-eight hours after transfection, the cells were collected. Then the gadd7 expression levels in pcDNA3.1-gadd7 or negative control plasmid transfected cells were analyzed. The relative expression levels of gadd7 in GC-1 and GC-2 cells treated with pcDNA3.1-gadd7 plasmids were significantly up-regulated by 22.05 ± 4.42 fold and 20.32 ± 3.59 fold, respectively. Data are presented as the mean ± standard deviation.

**Figure 2:**
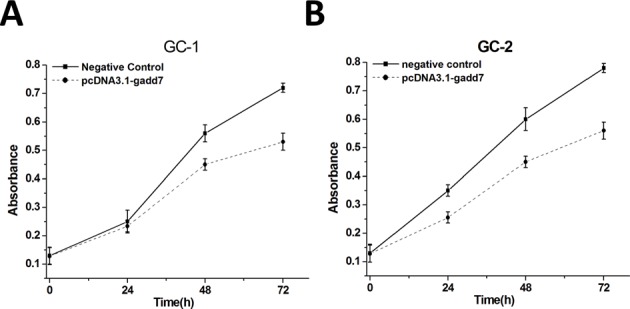
Cell proliferation changes caused by transfection of pcDNA3.1-gadd7 Cell proliferation was measured by MTT assay.pcDNA3.1-gadd7 inhibited GC-1 **(A)** and GC-2 **(B)** proliferation. Data are indicated as mean ± standard deviation. Each experiment in both cell lines was performed in triplicate for three independent times. MTT assays were analyzed using ANOVA.

To investigate the possible impact of gadd7 on the proliferation of GC-1 and GC-2 cells, the cell proliferation was determined by MTT assay. As shown in Figure 2, cell growth inhibition was obtained in GC-1 (Figure 2A) and GC-2 (Figure 2B). Significant differences were demonstrated for all time frames between the pcDNA3.1-gadd7 and negative control plasmid -transfected cells.

### Overexpression of gadd7 promoted cell apoptosis

**Figure 3:**
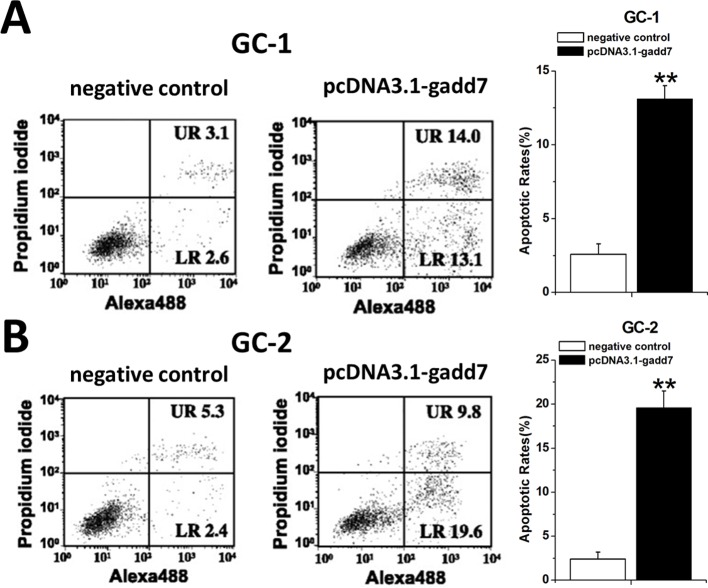
Involvement of gadd7 in cell apoptosis GC-1 and GC-2 cells were transfected with the plasmids in 6-well plates. Cell apoptosis was measured by the flow cytometry at 48 h post transfection. Representative images of flow cytometry analysis in GC-1 cells **(A)** and GC-2 cells **(B)** were shown. Cell apoptosis induction was observed in pcDNA3.1-gadd7 transfected GC-1 and GC-2 cells using flow cytometry analysis. Error bars, standard deviation. ^**^P<0.01, compared with the negative control. The apoptosis differences were analyzed using independent samples *t*-test.

To investigate the possible impact of gadd7 on the apoptosis of GC-1 and GC-2 cells, the cell apoptotic rates of these cells were determined using an Alexa488-PI apoptosis detection kit. The results shown in Figure 3 demonstrated that the apoptotic cells (%) of GC-1 (Figure 3A) and GC-2 (Figure 3B) cell lines transfected with the pcDNA3.1-gadd7 were higher than those transfected with the negative control plasmid.

### Overexpression of gadd7 increased Bax and reduced Bcl2 protein expression

**Figure 4:**
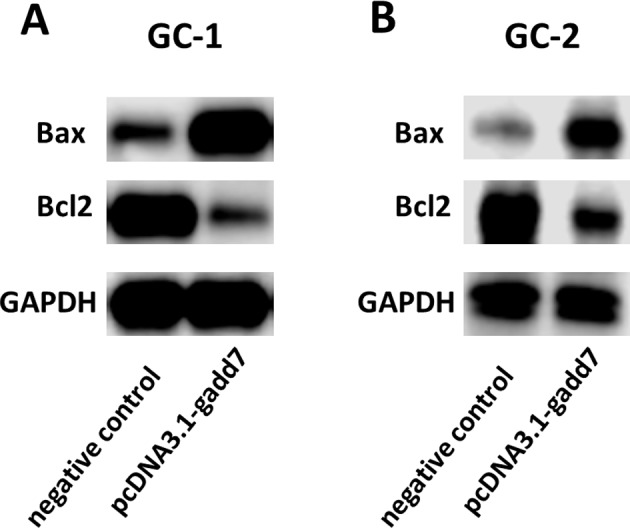
Overexpression of gadd7 increased Bax and decreased Bcl2 protein expression After transfection of pcDNA3.1-gadd7 or negative control, western blot assay was used to detect expression changes of Bax and Bcl2 in GC-1 and GC-2 cells. **(A)** Representative images of western blot assay in GC-1 cells. **(B)** Representative images of western blot assay in GC-2 cells.

To investigate the potential bio-markers that induce the above phenotypic changes after overexpression of gadd7, we used western blot assay to determine the protein levels of Bax and Bcl2 that are well-known for cell apoptosis. pcDNA3.1-gadd7 significantly up-regulated the expression of Bax and down-regulated Bcl2 at protein levels in GC-1 (Figure 4A) and GC-2 cells (Figure 4B).

## DISCUSSION

LncRNAs are emerging as endogenous triggers of the signaling pathways and play various functions in different human diseases. Recent studies have identified stress-induced miRNAs as potential biomarkers for varicocele [10–12]. These phenomena raise questions about the ability of lncRNAs to induce stress response, since the existing data point to the presence of a complex network of lncRNAs and miRNAs.

gadd7 is a DNA damage-inducible lncRNA, which controls the G1/S checkpoint in response to UV irradiation [13]. gadd7 binds to TDP-43 and promotes Cdk6 mRNA decay. Therefore, gadd7 induces the cellular response to DNA damage and overexpression of gadd7 leads to a decrease in hamster ovary (CHO) cell growth. gadd7 is also found to be a regulator of lipid-induced oxidative and endoplasmic reticulum stress [14]. However, its role in varicocele remains largely unknown.

In the present study, we found that gadd7 was up-regulated in varicocele group compared to normal healthy group. We further hypothesized that gadd7 may induce sperm impairment. To prove this possibility, we determine the possible impact of gadd7 on GC-1 and GC-2 cells, two commonly used cell lines for studying male infertility. Cell proliferation inhibition and increased apoptosis were observed in gadd7-transfected GC-1 and GC-2 cells. The western blot results suggest that stress-induced gadd7 may lead to cell death and male infertility via upregulating pro-apoptotic regulator Bax [15] and downregulating anti-apoptotic regulator Bcl2 [16] in varicocele patients. This work provides a potential novel insight for the varicocele-related sperm impairment and male infertility. In future works, the differential expression patterns of gadd7 may have implications in guiding diagnosis and treatment of varicocele.

## MATERIALS AND METHODS

### Clinical sample collection

56 patients with varicocele and 28 healthy donors with normal semen quality participated in the study. These varicocele sufferers were also infertile patients who had a history of ≥1 year of failed attempts to achieve a pregnancy after regular sexual activity. Exclusion criteria include seminal infection, cryptorchidism, orchitis, testicular atrophy and long-term smoking history. Varicocele was diagnosed according to international clinical classification guidelines: grade I, no visible or palpable distension except when the man performs the Valsalva maneuver; grade II, palpable but not seen; and Grade III, large enough to be visible. A Fresh ejaculate samples were collected from the patients and donors. Normal sperm parameters were defined according to World Health Organization (2010) criteria. The spermatozoa vitality was assessed by hypo-osmotic swelling test according to WHO criteria. The study was approved by the Institutional Review Board of Foshan Maternal and Child Health Hospital (Foshan, China) and written informed consents from all patients were obtained.

### Cell lines and cell culture

The mouse germ cell lines GC-1 and GC-2 were obtained from the Institute of Cell Research, Chinese Academy of Sciences, Shanghai, China. Cells were cultured in RPMI 1640 medium supplemented with 10% fetal bovine serum (Invitrogen) at 37°C in a 5% CO_2_ atmosphere.

### RNA extraction and real-time quantitative PCR

Total RNA was extracted from the sperm pellets using TRIzol (Invitrogen) according the manufacture's protocol. RNA was treated with RQ1 RNase-free DNase (Promega) to eliminate genomic DNA contamination and the cDNA strand was synthesized from total RNA with the RevertAidTM First Strand cDNA Synthesis Kit (Fermentas, Hanover, MD). The primer sequences were as follows: gadd7 forward primer: 5’- ACAATGACGCCATCGTTTTCT-3’, reverse primer: 5’-TGTCCTCCATCTGGGCATTT-3’; GAPDH forward primer:5’ -CGCTCTCTGCTCCTCCTGTTC-3’, GAPDH reverse primer: 5’ -ATCCGTTGACTCCGACCTTCAC-3’. PCRs were set up in a total volume of 20 μl, including 10μl of All-in-OneTMqPCR Mix (GeneCopoiea Inc., Rockville, MD), 0.4μl of forward primer, 0.4μl of reverse primer, 1μl of First-Strand cDNA, 0.4 μl of 50×ROX Reference Dye and 7.8μl of double-distilled water. The reactions were performed in the ABI PRISM 7000 Fluorescent Quantitative PCR System (Applied Biosystems, Foster City, CA). The PCR cycling parameters were: 95°C for 15 min, followed by 40 cycles of 94°C for 15 s, 55°C for 30 s and 72°C for 30 s. Expression fold changes were calculated using 2^−ΔΔCt^ methods.

### Plasmid construction and cell transfection

The gadd7 containing vector (gadd7 overexpression vector driven by CMV promoter) and the control vector (control vector) were purchased from GeneCopoiea Inc, Rockville, MD, USA. pcDNA3.1-gadd7 and the negative control were transfected into cells using Nanofectin™ Transfection reagent (Excell Bio, Shanghai, China) according to the protocol. The final concentration of plasmid was 1μg/ml.

### Cell proliferation assay

Cell proliferation was measured by 3-[4,5-dimethylthiazol-2-yl]-2,5-diphenyl-tetrazolium bromide (MTT) assay. Cells were grown in a 96-well plate for 24 h, transfected with pcDNA3.1-gadd7 or negative control and cultured in normal medium. Cells were then incubated in 0.1 mg/ml MTT at 37°C for 4-6 h and lysed in dimethyl sulfoxide (DMSO) at room temperature for 10 min at 0, 24, 48, and 72 h after transfection. The absorbance in each well was measured at 490 nm by a microplate reader (Bio-Rad, Hercules, CA). Each experiment was done at least three times.

### Flow cytometry assay

GC-1 and GC-2 cells were transiently transfected with plasmid vectors. 48 hours after transfection, cells were harvested and resuspended in fixation fluid. 5 μl of Annexin V – FIFC and 2 μl of propidium iodide were added to 500 μl of cell suspension. Cell apoptosis was then determined by using flow cytometry (EPICS, XL-4, Beckman, CA, USA). In the graphs, the quadrant respectively stands for dead cells, living cells, early apoptotic cells and late apoptotic cells.

### Western-blot assay

Cells were lysed in RIPA buffer (50 mM Tris-HCl pH 7.2, 150 mM NaCl, 1% NP40, 0.1% SDS, 0.5% DOC, 1 mM PMSF,25 mM MgCl2, and supplemented with a phosphatase inhibitor cocktail). Lysates from equivalent cell numbers were electrophoresed on SDS–polyacrylamide gels and transferred to PVDF membranes. After blocking with 5% milk, membranes were incubated with specific primary antibodies (Abcam, Cambridge, MA, USA) against Bax and Bcl2. Then, the blot was incubated with secondary antibody for one hour at room temperature on a rocking platform.

### Statistical analyses

Data analyses were performed by ANOVA or independent samples *t*-test, respectively, using the SPSS (Version 19.0 SPSS Inc.). P<0.05 was considered to be statistically significant.
